# A case report of heterotopic ossification of the tendon sheath after blunt trauma

**DOI:** 10.1097/MD.0000000000043723

**Published:** 2025-08-01

**Authors:** Kazuhiro Otani, Ryosuke Kakinoki, Koji Gotoh, Shuhei Oda, Mikio Nakajima, Mutsumi Ohue

**Affiliations:** a Department of Orthopedic Surgery, Faculty of Medicine, Kindai University, Osaka-Sayama, Osaka, Japan; b Department of Orthopedic Surgery, Katsuragi Hospital, Kishiwada, Osaka, Japan.

**Keywords:** case report, finger, flexor tendon, Heterotopic ossification

## Abstract

**Rationale:**

Heterotopic ossification of the hand is relatively rare. Herein we report a case of heterotopic ossification around the tendon sheath.

**Patient concerns:**

A 46-year-old man presented to the hospital with an inability to flex his right index finger, and with pain during motion. He had a history of blunt trauma to the right index finger during work. Clinical examination revealed a localized mass and tenderness in the volar aspect of the right index finger. Active flexion of the affected fingers was limited. No limitations on active finger extension and passive flexion were noted. Radiography and computed tomography revealed a ring-like bony lesion on the palmar aspect of the proximal phalanx that appeared to develop around the flexor tendon.

**Diagnoses:**

Constriction of the flexor tendons at the annular 2 pulley where heterotopic ossification occurred was thought to be the etiology of symptoms.

**Interventions:**

Complete resection of the ossified lesion and the distal half of the annular 2 pulley was performed. Postoperative recovery was uneventful.

**Outcomes:**

Six months after surgery, complete extension and flexion of the finger were evident, without symptoms.

**Lessons:**

This case report demonstrates that heterotopic ossification can occur at the tendon sheath of the finger. Heterotopic ossification at the flexor tendon sheath may result in the limitation of finger movement. Radiographic examinations are helpful to confirm the presence of osseous lesions in cases with suspected heterotopic ossification. Complete excision of ossified lesions improves symptoms.

## 1. Introduction

Heterotopic ossification is the abnormal formation of bone in the extremities, and it commonly affects the hip, shoulder, and elbow joints. It generally occurs around a joint, and surgical treatment may be required in cases of limited range of motion or pain on movement of the affected joint. The hand is a rare site for heterotopic ossification.^[1]^ Herein we report a case of heterotopic ossification in the tendon sheath that caused entrapment of flexor tendons, in which the patient underwent successful complete resection of the ossified lesion.

## 2. Case presentation

A 46-year-old right hand-dominant man presented to our hospital with an inability to flex his right index finger, and pain when attempting to move that finger. He had experienced blunt trauma to the right index finger at work 6 months prior. Neither scarring nor swelling was observed. Symptoms had resolved spontaneously after the trauma incident, and he had not undergone any medical treatment. Two months after the trauma, he experienced pain during motion of the finger, as well as limited flexion, and visited our orthopedic department.

**Figure 1.**
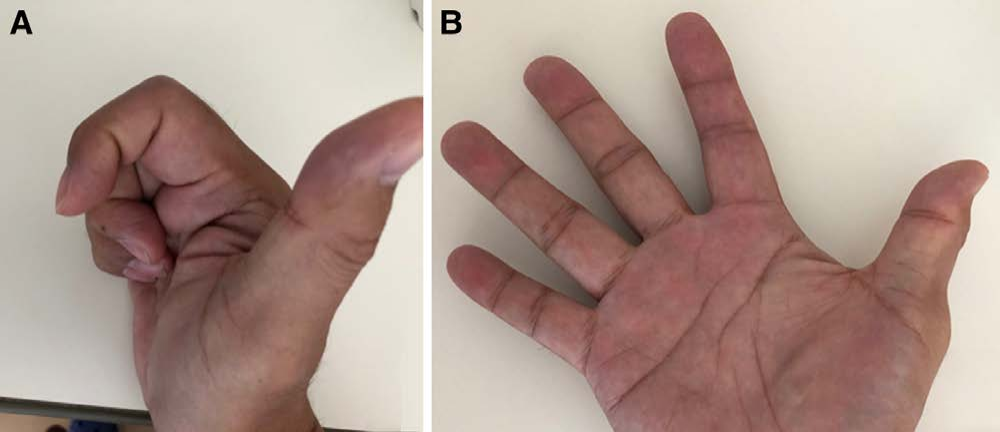
Clinical photographs of the hand. (A) Active extension. (B) Active flexion.

Clinical examination revealed a localized mass and tenderness in the volar aspect of the right index finger. Active flexion angles of the right index finger were restricted to 40° in the metacarpophalangeal joint, 80° in the proximal interphalangeal joint, and 45° in the distal interphalangeal joint, whereas passive flexion was normal (Fig. 1). No limitation of active finger extension was noted. The patient did not experience snapping.

**Figure 2.**
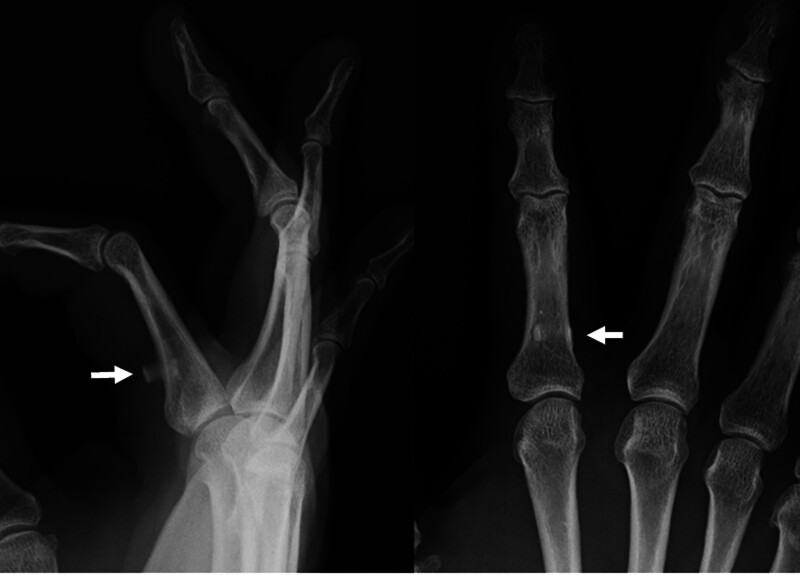
Radiographs of the right hand. Arrows indicate heterotopic ossification.

**Figure 3.**
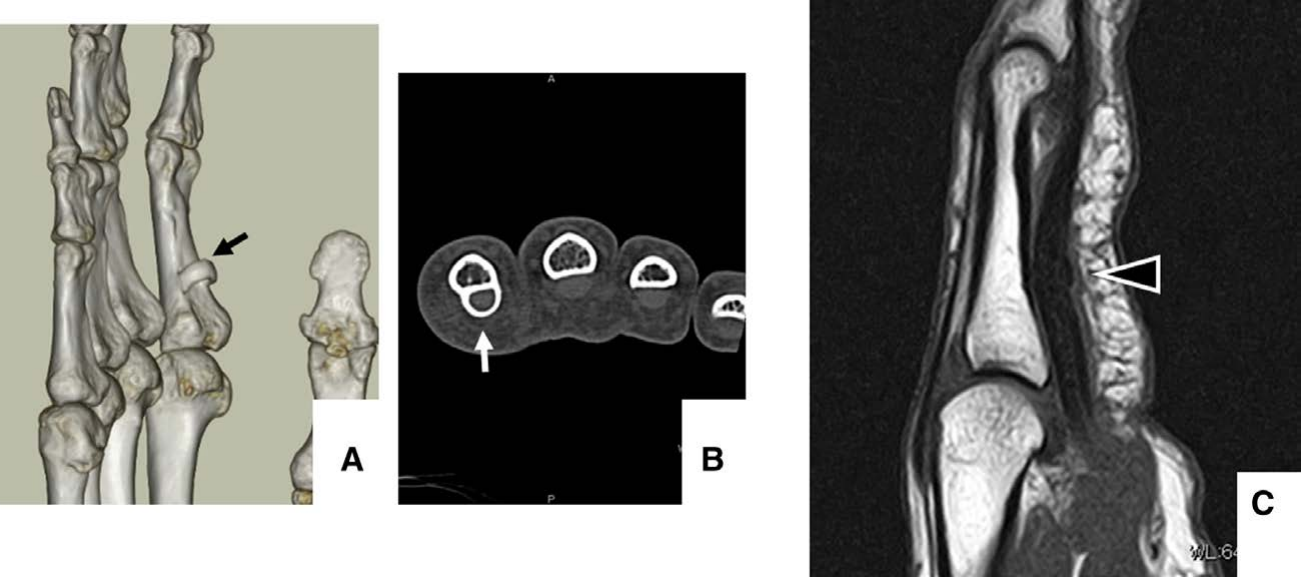
Computed tomography (CT) and magnetic resonance imaging (MRI) findings of the right index finger. (A) Three-dimensional CT and (B) two-dimensional CT indicated heterotopic ossification (arrows) around the tendon sheath. (C) MRI results. Arrowheads indicate ossification constricting the flexor tendon. Arrows indicate heterotopic ossification around the tendon sheath.

Radiography revealed an osseous lesion in the volar aspect of the proximal phalanx (Fig. 2). Two-dimensional computed tomography and three-dimensional computed tomography revealed a ring-like bony lesion on the palmar aspect of the proximal phalanx that appeared to develop around the flexor tendon (Fig. 3A, B). Magnetic resonance imaging indicated entrapment of the flexor tendons at the bony lesion (Fig. 3C). It was therefore concluded that an ossified flexor tendon sheath constricted the flexor tendon, and caused limitation of flexion of the index finger. No preoperative medication or local injections were administered because the bony lesion was thought to be the cause of the flexion impairment in the finger.

**Figure 4.**
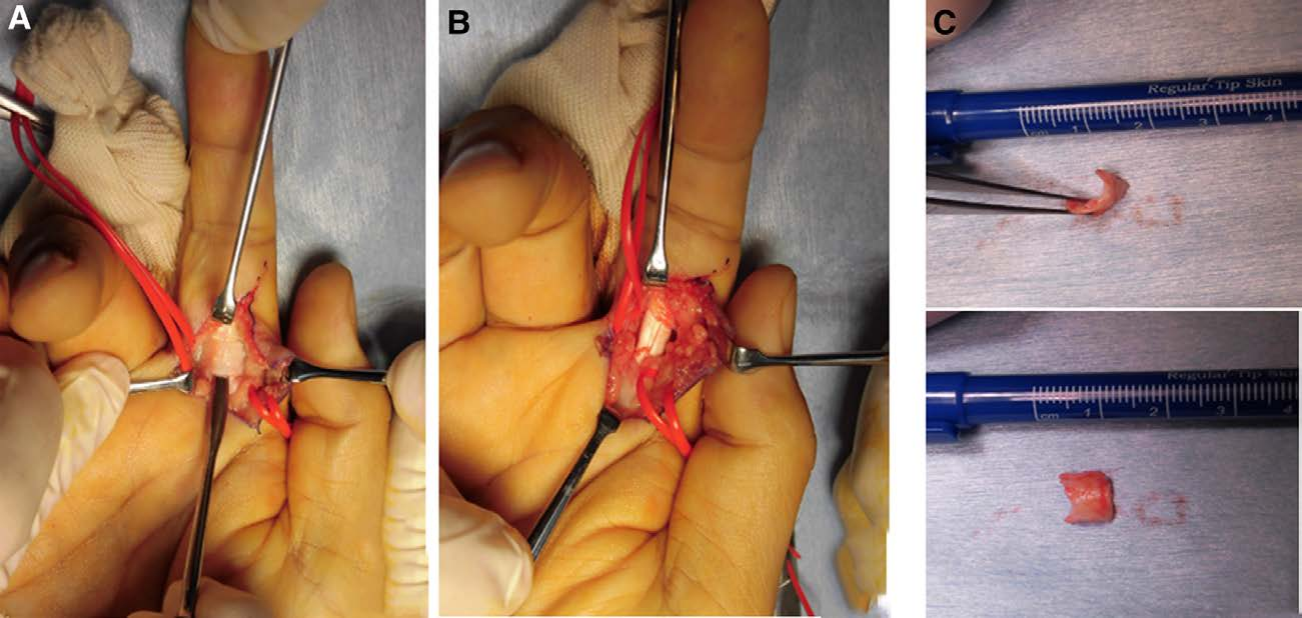
Intraoperative photographs of the right index finger. (A) Heterotopic ossification of the A2 pulley. (B) After resection of the heterotopic ossification. (C) Resected tissue. A2 pulley = annular 2 pulley.

**Figure 5.**
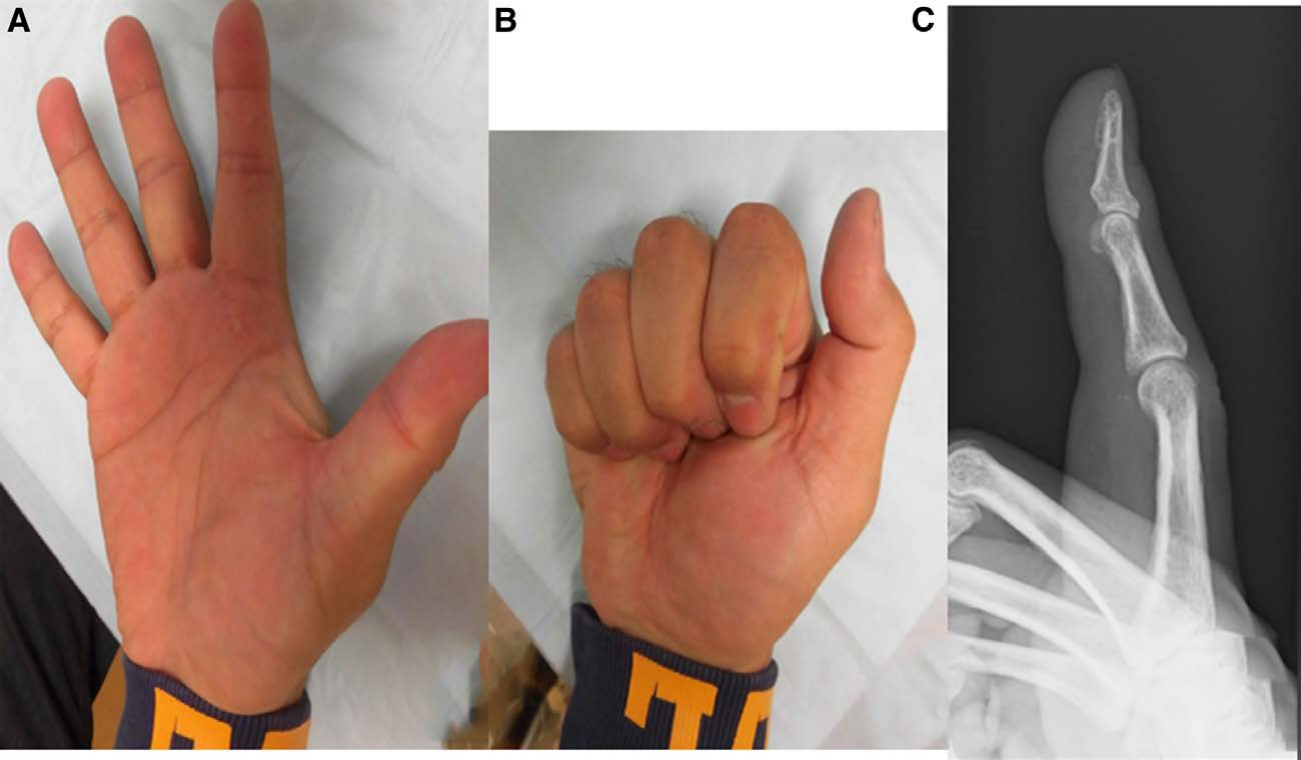
Clinical photographs of the hand after surgery. (A) Active extension. (B) Active flexion. (C) Postoperative radiography.

Complete resection of the ossified lesion was performed using the volar Bruner approach 3 months after onset. Neurovascular structures were preserved. An osseous lesion was located in the distal half of the annular 2 pulley (A2 pulley). The flexor tendons were constricted at the lesion. The proximal part of the A2 pulley was carefully preserved, and the osseous lesion was removed, accompanied by the distal part of the A2 pulley (Fig. 4). Postoperatively the patient was treated with oral nonsteroidal anti-inflammatory drugs for 1 month to prevent the recurrence of heterotopic ossification. Postoperative recovery was uneventful. At a follow-up visit 6 months after the surgery he reported no pain or tenderness in his right index finger, ranges of active and passive motion had increased to normal levels (Fig. 5), he had fully returned to his previous work, and he was satisfied with his treatment.

## 3. Discussion

Heterotopic ossification may occur as a post-trauma complication. It often occurs around the large joints (e.g., hip, shoulder, and elbow) and causes limitation of range of motion. Heterotopic ossification is thought to have 5 potential causes; genetic, post-traumatic, neurogenic, postsurgical, and reactive lesions.^[2]^ Reactive lesions sometimes occur in the hand. Reactive ossifying lesions of the hand include Nora lesions, florid reaction periostitis, and subungual exostosis. Because these reactive lesions are thought to be associated with the periosteum or periarticular surrounding tissue, they are not regarded as heterotopic ossifications.

Two mechanisms have been proposed for heterotopic ossification formation. One hypothesis is that repetitive motion or early mobilization causes accumulated mechanical stress and leads to ossification in the surrounding joint. The second hypothesis is that trauma causes increased expression of bone morphogenic proteins that play a role in ectopic ossification. The current patient experienced blunt trauma. No bone or soft tissue injuries were observed. Hemorrhage in the soft tissue surrounding the tendon sheath after blunt trauma may lead to bone formation in the tendon sheath. In previous reports ectopic calcification of the fingers was found in subcutaneous tissue. Unlike previously reported cases, the present case is a rare instance of heterotopic ossification arising from the tendon sheath.

There are some previous reports of heterotopic ossification arising in the hand.^[3–9]^ Of these, 3 involved heterotopic ossification after a head or spinal cord injury,^[3–5]^ 1 after a surgical procedure,^[6]^ 1 after a local steroid injection,^[7]^ 1 after blunt trauma,^[8]^ and 1 after a flexor tendon injury.^[9]^ These previously reported cases are summarized in Table 1. The current patient exhibited heterotopic ossification after closed blunt trauma of the finger. Most patients in the aforementioned reports exhibited heterotopic ossification arising from the soft tissue, joint capsule, or phalangeal bone. The present patient experienced blunt trauma to the right index finger without any bony or articular injuries. Lorio and Duplechain^[9]^ described a case in which heterotopic ossification developed on the volar surface of the phalangeal bone, which is similar to the present case. However, we could not locate any previous reports of heterotopic ossification of tendon sheaths. Neither laceration nor scar formation were observed in the tendon sheath during surgery. We speculate that the hematoma that formed around the flexor tendon sheath after the blunt trauma may have developed into an ossified lesion.

**Table 1:** Previously reported cases of heterotopic ossification of the finger.

References	Age (years)	Gender	Side	Cause	Site of the lesion	Location of the finger	Location of the bone	Management
Bendeddouche et al^[3]^	47	M	Bilateral	Spinal cord injury	2nd, 3rd, 4th	Proximal phalanx	Soft tissue	NSAID
Meythaler et al^[4]^	44	M	Right	Spinal cord injury	2nd, 3rd, 4th	Proximal phalanx	Extensor tendon	NSAID
Spencer^[5]^	45	M	Left	Head injury	3rd	PIP joint	Volar capsule	None
Barlaan et al^[6]^	57	M	Right	Tendon and nerve injuries	3rd	Proximal phalanx	Soft tissue	Surgical
Li et al^[7]^	37	M	Right	Steroid injection	3rd	Proximal phalanx	Soft tissue	NSAID
Tan et al^[8]^	27	M	Right	Blunt trauma	2nd	Proximal and middle phalanx	Soft tissue	NSAID
Lorio et al^[9]^	33	M	Left	Tendon injury	2nd	Proximal phalanx	Bone surface	Surgical
Current case (2025)	46	M	Right	Blunt trauma	2nd	Proximal phalanx	Flexor tendon sheath	Surgical

Treatments for heterotopic ossification can be categorized as either conservative or surgical. Conservative treatments include nonsteroidal anti-inflammatory drugs, radiation, oral etidronate therapy, and physiotherapy. Heterotopic ossification generally occurs around joints, thus surgical treatment may be considered in cases of limited range of motion or pain during motion due to heterotopic ossification in periarticular lesions. Furthermore, surgical treatment is usually delayed until heterotopic ossification reaches mature bone because of the possibility of recurrence after surgical excision. Surgery is generally performed 6 months after trauma, 1 year after spinal cord injury, and 1.5 years after brain injury.^[10]^ In small joints such as those in fingers, long-standing limitations of the range of motion may cause tendon and joint contracture. Barlaan and Wing-Yuk^[6]^ suggested that the appropriate time for excision of heterotopic ossification in the finger differs from that in larger joints such as the hip, elbow, and shoulder. Surgical resection was performed 9 months after trauma in the current patient because active flexion of the finger was prohibited due to flexor tendon entrapment at the A2 pulley. Ossification was localized to the tendon sheath and constricted the flexor tendons; therefore, complete resection was performed to resolve the symptoms. No recurrence was observed 1 year after surgery. In clinical practice we often encounter patients who develop motor disturbance in a finger (e.g., snapping finger, giant cell tumor of tendon sheath, and flexor tendon injury). Most of these phenomena can be diagnosed without the need for diagnostic imaging, except in the case of tumors. It is important to consider that heterotopic ossification may occur after minor trauma and can result in the limitation of finger movement. Radiographic examinations are essential to confirm the presence of osseous lesions in cases of suspected heterotopic ossification.

## Acknowledgments

We thank Editage (www.editage.jp) for English language editing.

## Author contributions

**Formal analysis:** Kazuhiro Otani.

**Methodology:** Shuhei Oda.

**Project administration:** Mikio Nakajima, Mutsumi Ohue.

**Supervision:** Ryosuke Kakinoki, Koji Gotoh.
